# On the Operation for Cleft Palate

**Published:** 1853-04

**Authors:** John Gay

**Affiliations:** Surgeon to the Royal Free Hospital.


### ARTICLE XXIII.

*On the Operation for Cleft Palate.* By John Gay, Esq., F. R. C. S., Surgeon to the Royal Free Hospital.

Charlotte D——, aged twenty, a delicate-looking girl, was admitted into the Royal Free Hospital on December 9th of last year, having a congenital cleft of the hard and soft palates, and an imperfectly-united hare-lip, with a redundancy of substance in the lip itself. Mr. Gay removed the cicatrix and re-united the lip, with considerable advantage to the patient, and she was allowed to return home for a short period.

On January 11, she was re-admitted, in order to have the soft palate united, preparatory to the use of an obturator. The fissure of the hard palate was three quarters of an inch in breadth at its posterior part, diminishing somewhat anteriorly, where it tended to the left side, and terminated at a part corresponding to the interval between the left lateral incisor and canine teeth. The sides of the velum were healthy, and presented the usual muscular resistance on being drawn out. Deglutition was performed with greater ease than in some cases of cleft of the soft palate only, but her speech was almost unintelligible, and she was on that account exceedingly anxious to have the operation performed.

On January 21, Mr. Gay adopted the proceeding recommended by Mr. Fergusson, and which he has usually practiced in similar cases—viz. section of the levatores palati and palato-pharyngei muscles, in order to relax the palate by depriving it of muscular action. This enabled the posterior valves of the velum to be approximated without difficulty; but the width of the cleft in the hard palate would occasion considerable tension of the flaps at their anterior part when brought together in consequence of the firm muscular and fibrous connexion of the velum with the posterior edge of the transverse plates of the ossa palati. To prevent such strain on the velum, Mr. Gay, in this case, as in the two others detailed, made a transverse incision immediately behind the hard palate; this had the desired effect, and the edges of the fissure having been previously pared, the parts were united by three sutures.

During the course of the operation, which was rather more tedious than usual, in consequence of the smallness of the patient's mouth, and the usual depth of the soft palate, the parts bled rather freely; but the hemorrhage was easily arrested by the use of ice water before the stitches were tied.

The inflammation following was of a healthy character; union by the first intention had taken place after four days, and two of the sutures were removed; the remaining one was removed on the fifth day, when union was complete throughout.

From the first she was allowed a necessary amount of nourishment; during the first two days, as there was some difficulty in swallowing, and she could only take a little arrow root by the mouth, she was ordered a pint of strong beef-tea, with a glass of sherry, as an enema.

On the third day the bowels were freely relieved by a purgative draught. The palate was cleansed daily, and lightly sponged with a little myrrh lotion, which was accomplished without producing any pain.

The transverse incisions were scarcely visible by the time the sutures were removed, the divided parts having yielded to the increased breadth of the united velum.

As soon as the palate has regained its healthy tone, an obdurator will be adapted to the cleft in the hard palate, thus completing the case.

Mary B——, aged twenty-one years, admitted under Mr. Gay's care, October 8, suffering from congenital cleft of both hard and soft palates, complicated with hare-lip and fissure of the alveolar arch, the division of the latter corresponding to the interval between the lateral incisor and canine teeth on the left side; there was also an irregular disposition of the front teeth, some of which projected in various directions; others were only partially developed, and bounded the sides of the cleft. The margins of the cleft were pared, and four sutures were introduced. The bleeding was inconsiderable, and had stopped before the stitches were tied. From the first, nutrient fluids were administered frequently and in small quantities. After five days, the parts having united, all but a very small piece in the middle, the stitches were removed, and the small aperture left closed subsequently by granulations.

Miss W——, of Manchester, aged sixteen. This young lady was afflicted with a cleft, extending, as in the other two cases, through the hard and soft palate; indeed, the hard palate was almost entirely wanting, being limited to a slight ridge on either side. The distance between the edges of the imperfectly-developed horizontal plates of the palate bones was one inch and an eighth; the distance between the anterior molar teeth being one inch and four-tenths only. Owing to the absence of the lateral incisors, without any cleft of the alveolar arch itself, the alveolar processes of the superior maxillary bones took a straight instead of a curved course from behind, to their point of union anteriorly; from this condition of parts the fissure in the hard palate was angular, and diminished rather rapidly from behind forwards. The defect was one of great advantage to the young lady, and the question to be decided was, whether the soft flaps could be made to unite through the whole length, so that a gold obturator might supply the remaining deficiency. Mr. Fergusson saw this case, and doubted its practicability, owing to the extreme distance by which the soft flaps were separated. Mr. Gay determined, however, to make the trial, and adopted the method recommended by Mr. Fergusson, but in addition practiced the transverse incisions before alluded to; by this means the flaps were released, and could be brought together mesially without tension. Four sutures were employed, and they were removed on the fourth day. This case was eminently successful, and an obturator was supplied, admirably adapted to the case, by Mr. Tibbs, the dentist of Finsbury-place, which has been worn since with the greatest comfort.

---

The three cases, detailed above by Mr. Lane, illustrate some important points in reference to the operation for cleft palate. The labors of Mr. Fergusson have rendered the treatment of these cases all but perfect, the remaining defect being limited to the frequent occurrence of a small aperture, which has to a certain degree compromised the value of the operation. I refer to the aperture which has very frequently remained between those portions of the edges of the flaps in immediate proximity to the tubercle of the hard palate, the edges of which will, on examination, be observed to be thinned off, and generally of a hard and fibrous character.

These apertures, after the healing of the remainder of the flaps, are of various sizes; in *some* cases they go on to closure, but their completely closing depends on two circumstances—first, the size of the aperture; and secondly, the general tension on the flaps; for, as was observed by Mr. Lane, at the Royal Free Hospital, these apertures heal or aproximate towards closing, not so much by addition to the edges through the medium of fresh tissue, as by a stretching of the flaps generally towards the axis of the opening. In other words, the apertures are mainly closed at the expense of the flaps, and by an increase of their general tension; the result of which is, that when the call on that tension has been obeyed to the utmost, the power of further closing ceases.

It is desirable, then, to give increased freedom to the flaps, and especially at that part where their freedom is interfered with by their connection with the bony palate.

When cases were presented to me in which the cleft extended itself throughout the bony as well as the soft palate, the question arose whether the latter could be united through its entire length; for on that being accomplished, a small obturator could easily be made to supply the deficiency in the hard palate, in case Mr. Avery's ingenious plan of closing it should not be feasible. The view ordinarily accepted—that in these cases there is no deficiency of soft parts; that where even the bony palate is limited to the merest ridge, still the soft palate is normally developed, although retained back by the deficient bony palate; moreover, that in children the soft palate has been known to become cleft after birth by a strain upon it in the act of crying, &c.,—led me to adopt the course that was successfully taken in these cases.

The results have shown, that as soon as the flaps are freed from connexion with the bone by *transverse incisions*, they unfold, and can be brought with the greatest ease into contact mesially.

It follows, then, that these transverse incisions, if made, but to a less extent, in the usual operation for a cleft of the *soft* palate only, will, by relieving the tension on the front part of the flaps, effectually prevent the defect alluded to—viz. the aperture which often remains an unworthy stigma on one of the most admirable and effective operations in surgery.

Moreover, these additional incisions will, to a certain degree, prevent that tension of the flaps which has been frequently observed to remain after their re-union by operation; and thus to render the palate better adapted for the performance of its functions, by making it more amenable to the muscular influences which act upon it.

